# Zinc Phosphide Poisoning: From A to Z

**DOI:** 10.3390/toxics11070555

**Published:** 2023-06-25

**Authors:** Anabell Juárez-Martínez, Jesús del Carmen Madrigal-Anaya, Yessika Paola Rodríguez-Torres, Ramsés Dorado-García, Daphne Marisol Montes-Ventura, Ahgiel Jiménez-Ruiz

**Affiliations:** 1 Department of Toxicology, Hospital Juárez de México, Mexico City 07760, Mexico; jcma_78@hotmail.com (J.d.C.M.-A.); pao_rguez@hotmail.com (Y.P.R.-T.); ramses.dorado@gmail.com (R.D.-G.); marycc_9@hotmail.com (D.M.M.-V.); 2 Intensive Care Unit, Hospital General la Perla, Mexico City 57830, Mexico; ahgiel@icloud.com

**Keywords:** zinc phosphide poisoning, phosphine, personal protective equipment, coconut oil, antioxidants, magnesium sulphate, hyperinsulinemia–euglycemia therapy, intravenous lipid emulsion

## Abstract

Zinc phosphide is a rodenticide that is used in agricultural, urban and industrial environments in México. After ingestion, it reacts with hydrochloric acid, hydrolyzing into phosphine. It causes cellular hypoxia via mitochondrial toxicity, resulting in multiple organ dysfunction and death. There is no antidote or specific treatment for zinc phosphide toxicity. We present the case of a 45-year-old female who ingested zinc phosphide with suicidal intent. On arrival at the emergency department, she had multisystemic disorders. Supportive care, decontamination and antidotal therapy were initiated. Subsequently, she evolved to clinical improvement with a resolution of the biochemical abnormalities of tissue hypoperfusion. She was discharged on day 7 without complications. In this review, we provide updated therapeutic options and discuss their specific pathophysiological basis.

## 1. Introduction

Zinc phosphide is a rodenticide that has been used since 1940 in agricultural, urban and industrial environments [1,2]. Since 1985, there are specific restrictions in the Regulations, Guidelines and Standards on its use as a pesticide. It is even prohibited in several countries. In Mexico, zinc phosphide is not prohibited or restricted for importation, manufacture, formulation, commercialization or use [3,4], this can be consulted in the search engine of the web site “Consultation of Sanitary Registers of Pesticides, Plant Nutrients and MRLs”, dependent on the governmental web site “Sanitary Register of Pesticides and Plant Nutrients” [5]. This makes it accessible to the general population, and although a cause–effect relationship between its availability and its use for suicidal purposes has not been reported, its accessibility makes it an easy xenobiotic to obtain, regardless of the purpose for which it is used.

When zinc phosphide comes into contact with water or acid and undergoes hydrolysis, it releases phosphine gas, which is the active component [1,2,6,7,8]. Zinc phosphide has a high mortality risk for humans, and thus, safe work practices must be taken during its production, storage and use [4]. In the current scientific literature, an intake of 80 mg/kg [9,10] was proposed as a lethal dose 50 for zinc phosphide in humans and a toxic dose range of 4 to 5 g total intake [5,11,12,13]. The most clinically relevant route of exposure to zinc phosphide in toxicology is the gastrointestinal route, which, upon contact with hydrochloric acid, releases phosphine via hydrolysis. Other routes of contamination with less clinical relevance were described, such as the cutaneous and ocular routes, which only manifest themselves through local irritation. The respiratory route has also been described as a means of absorption of phosphine that has already been released or of zinc phosphide dispersion products; however, both of these occurrences are low and their toxic potentials are of less clinical relevance and can be reduced with safe work practices [4,8]. The active component (phosphine gas) inhibits cytochrome C oxidase in complex IV of the mitochondrial respiratory chain, causing a disruption of oxidative phosphorylation [2,6]. It rapidly produces multi-organ cellular anoxia, increased formation of free radicals, breakdown of the ionic barrier, cell injury with irreparable damage to ribonucleic acids, and thus, cell death [1]. The lethality is attributed to heart failure [6,14,15]. A wide range of therapeutics is described in the medical literature, with different purposes, including reducing the absorption of the xenobiotic with gastric lavage with coconut oil, potassium permanganate, bicarbonate and the administration of activated charcoal after exposure via the gastrointestinal tract; hemodynamic support with intravenous solutions and vasopressors; innovative antidotal proposals, such as hyperinsulinemia–euglycemia therapy (HIET) and intravenous lipid emulsion (ILE), as well as counteracting oxidative damage with magnesium sulfate and N-acetylcysteine (NAC). None of these options are specific and they currently have up to limited efficacy [1,16,17,18].

We present the clinical case of a 45-year-old woman who ingested zinc phosphide with suicidal intent. An analysis of the therapeutics used and a review of the scientific literature on the subject are presented.

## 2. Case Description

**Figure 1:**
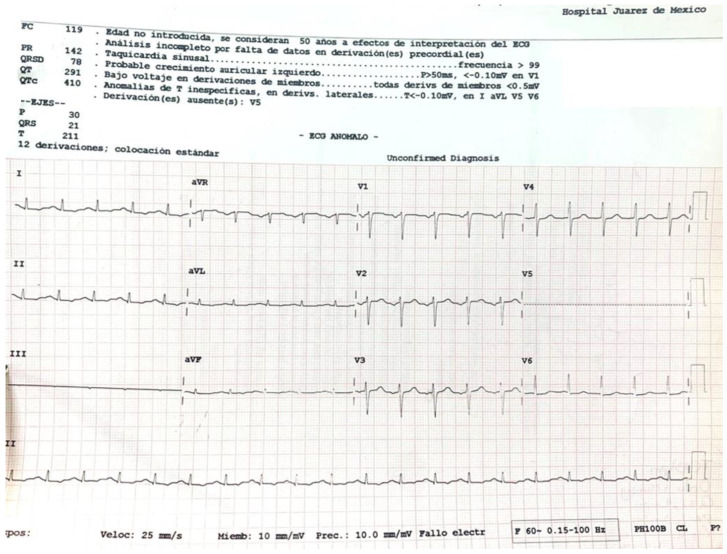
Electrocardiogram with sinus tachycardia. Sinus tachycardia is the most typical electrophysiological abnormality within the first 3 to 6 h, followed by ST-segment and T-wave changes [7]. Electrocardiogram obtained from the patient on admission to the Emergency Department of the Hospital Juarez de Mexico (Source: clinical record of the Hospital Juarez de Mexico).

A 45-year-old female with a history of untreated depressive disorder was admitted to the emergency department 6.5 h after ingesting 6 to 9 g of zinc phosphide (calculated weight dose of 80–120 mg/kg). She was found at her home in a supine position with indifference to the environment, with the presence of involuntary uresis and emesis of black content. A 50 g container of 24% zinc phosphide was found next to her. Later, she reported that she had consumed 500 cc of beer and, subsequently, three-quarters of the contents of the bottle of zinc phosphide. Upon admission to the emergency department, she was awake and reported nausea, vomiting, epigastric pain and anxiety. Her vital signs were as follows: blood pressure 94/74 mmHg, heart rate 129 beats per minute, respiratory rate 26 per minute, temperature 36.5 °C, SaO_2_ 87% (FiO_2_ 21%) and a FOUR scale score of 16 points. The clinical toxicology service was notified immediately. Electrocardiogram (Figure 1): sinus tachycardia, arterial blood gases with pH 7.298, pCO_2_ 22.2, pO_2_ 25.3, carboxyhemoglobin (COHb) 0.6, methemoglobin (MetHb) 1.1, HCO3 10.5, EB −14.3 and lactate 7.7.

**Figure 2:**
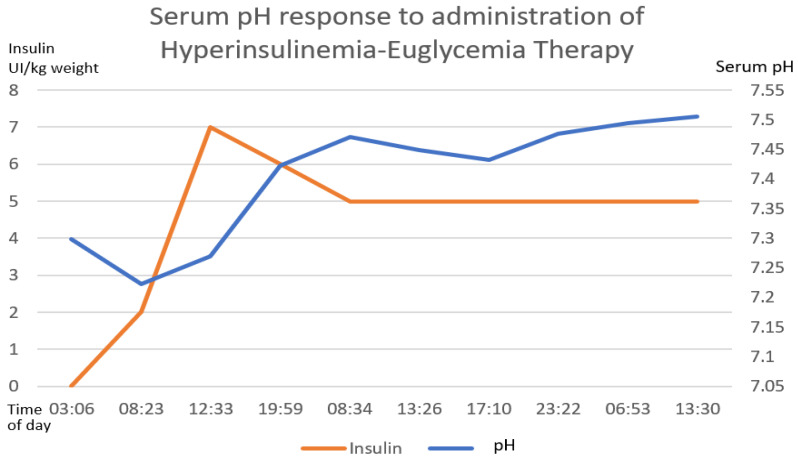
The direct correlation between the establishment of hyperinsulinemia–euglycemic therapy with the increase in serum pH until reaching normal ranges of serum pH. The first 58 h of treatment are graphed. The numerical scale and units of measurement of insulin, illustrated with the orange line, are plotted on the left-hand *y*-axis. The numerical scale and units of measurement of serum pH, illustrated with a blue line, are plotted on the right-hand “*y*”-axis.

**Figure 3:**
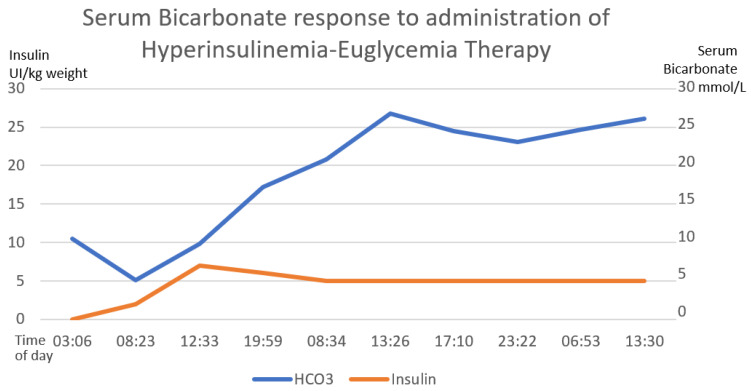
The direct correlation between the establishment of hyperinsulinemia–euglycemic therapy with the increase in serum bicarbonate until reaching normal ranges of serum bicarbonate. The first 58 h of treatment are graphed. The numerical scale and units of measurement of insulin, illustrated with the orange line, are plotted on the left-hand *y*-axis. The numerical scale and units of measurement of serum bicarbonate, illustrated with a blue line, are plotted on the right-hand “*y*”-axis.

**Figure 4:**
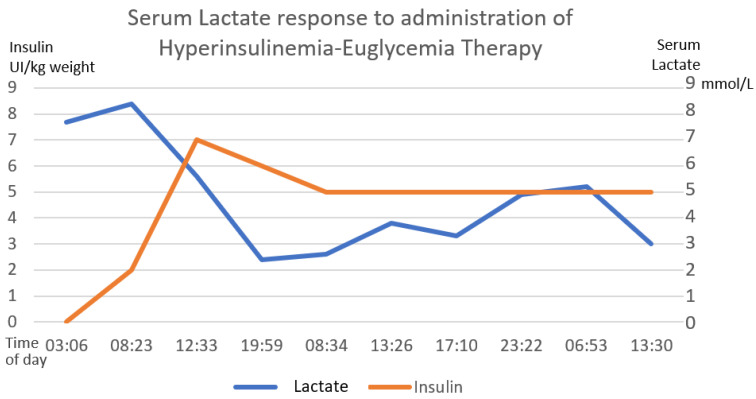
The inverse correlation between the establishment of hyperinsulinemia–euglycemic therapy with the decrease in serum lactate until reaching normal ranges of serum lactate is shown. The first 58 h of treatment are graphed. The numerical scale and units of measurement of insulin, illustrated with the orange line, are plotted on the left-hand *y*-axis. The numerical scale and units of measurement of serum lactate, illustrated with a blue line, are plotted on the right-hand “*y*”-axis.

**Figure 5:**
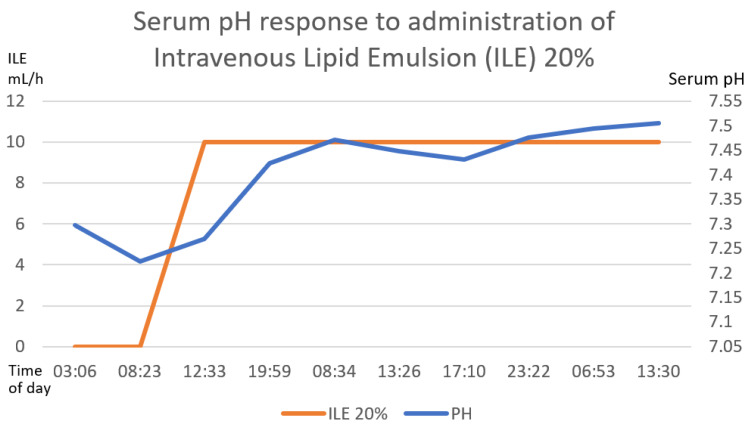
The direct correlation between the establishment of intravenous lipid emulsion 20% with the increase in serum pH until reaching normal ranges of serum pH. The first 58 h after treatment are graphed. The numerical scale and units of measurement of ILE 20%, illustrated with the orange line, are plotted on the left-hand *y*-axis. The numerical scale and units of measurement of serum pH, illustrated with a blue line, are plotted on the right-hand “*y*”-axis.

**Figure 6:**
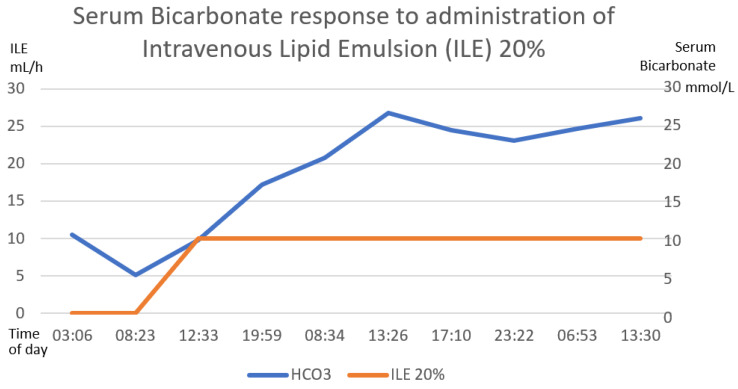
The positive correlation between the establishment of the intravenous lipid emulsion 20% and the increase in serum bicarbonate is shown. The first 58 h after treatment are graphed. The numerical scale and units of measurement of ILE 20%, illustrated with the orange line, are plotted on the left-hand *y*-axis. The numerical scale and units of measurement of serum bicarbonate, illustrated with a blue line, are plotted on the right-hand “*y*”-axis.

**Figure 7:**
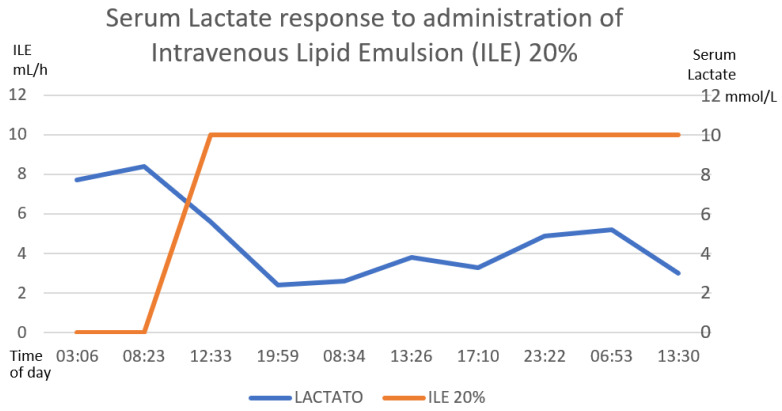
The inverse correlation between the establishment of the intravenous lipid emulsion 20% and the decrease in serum lactate is shown. The first 58 h after treatment are graphed. The numerical scale and units of measurement of ILE 20%, illustrated with the orange line, are plotted on the left-hand *y*-axis. The numerical scale and units of measurement of serum lactate, illustrated with a blue line, are plotted on the right-hand “*y*”-axis.

Gastric lavage was performed upon admission (7 h post-ingestion) with coconut oil (200 mL) plus bicarbonate (7.5% 50 mL). Continuous cardiac monitoring, request for laboratory studies and administration of crystalloid solutions were performed. She did present metabolic acidosis with a high anion gap with worsening for the first 6 h. The hyperinsulinemia–euglycemia therapy was initiated eleven hours after xenobiotic ingestion (Figure 2, Figure 3 and Figure 4), with a loading dose of 1 IU/kg/h and a maintenance dose of 1 IU/kg/h, requiring doses up to 7 IU/kg/h and a glucose contribution calculated at 0.5 g/kg/h, with a duration of therapy of 78 h. The serum glucose supply was withdrawn 21 h after insulin withdrawal. Magnesium sulfate was also administered at a dose of 1 g per hour for 24 h, then 1 g every 6 h for 4 days. N-acetylcysteine was given orally at a loading dose of 140 mg/kg and a maintenance dose of 70 mg/kg for 17 doses, always diluted in saline solution and 7.5% sodium bicarbonate with a 1:1 ratio. Treatment was provided with 20% lipid emulsion (Figure 5, Figure 6 and Figure 7) 13 h after ingestion of the poison, with a loading dose of 1.5 mL/kg, a maintenance dose of 10 mL/h for 24 h and a therapeutic duration of 84 h. Furthermore, low-flow oxygen therapy, an antiemetic and a proton pump inhibitor were given, with monitoring and management of hydroelectrolytic disorders.

Nine hours after the treatment was started, she showed clinical and biochemical improvement (Figure 2, Figure 3, Figure 4, Figure 5, Figure 6 and Figure 7), and 16 h later, there were no signs of tissue hypoperfusion or hemodynamic instability (without requiring vasopressor support or bicarbonate replacement). She was discharged 7 days later without complications, with follow-up by psychology and psychiatry.

## 3. Discussion

Zinc phosphide is colorless, odorless, flammable and highly toxic; when it comes into contact with water or hydrochloric acid in the stomach [1,14], it quickly hydrolyzes and emits phosphine gas (PH3) [6] (between 6.5 and 21 min is the time it takes to produce a large volume of lethal phosphine) [2]. It is absorbed through the mucous membranes via simple diffusion [2], inhalation, ingestion, or contact with the skin or mucous membranes [2,19,20,21,22], and is excreted mainly through the kidneys and lungs [2]. Generally, symptoms of toxicity develop within the first 15 min [19], causing nausea, vomiting, dyspnea and abdominal pain. Refractory hypotension, heart failure, cardiotoxic shock and severe metabolic acidosis lead to the death of the patient [1,16]. Most deaths occur within the first 12 to 24 h, usually due to cardiovascular collapse [19].

The mechanisms of toxicity are not clear, but several were described. Cellular hypoxia caused [6,14,19] by non-competitive inhibition of cytochrome C oxidase [23], which is characterized by mitochondrial toxicity, interferes with the transfer of electrons between the complexes II to IV of the respiratory chain, resulting in ATP depletion, oxidative stress and the release of free radicals [14,17]. This mechanism results in lipid peroxidation, which causes cell injury, and oxidative mechanisms, which cause protein denaturation and cell membrane dysfunction [1,17] due to catalase depletion, peroxide dismutase and glutathione reduction [6,14]. By inhibiting cytochrome C oxidase in cardiac cells, phosphine negatively affects mitochondria [7] and myocardial proteins. Alterations in myocardial mitochondria and proteins produce alterations in the permeability of sodium, potassium, magnesium, calcium and other ions, causing changes in the transmembrane action potential [14,19]. Heinz bodies were also observed, with changes in the valence of the heme component of hemoglobin. In vivo reports show intravascular complications, such as hemolysis and methemoglobinemia, which are typically suspected with an “Oxygen Saturation Gap” [7,14].

**Figure 8:**
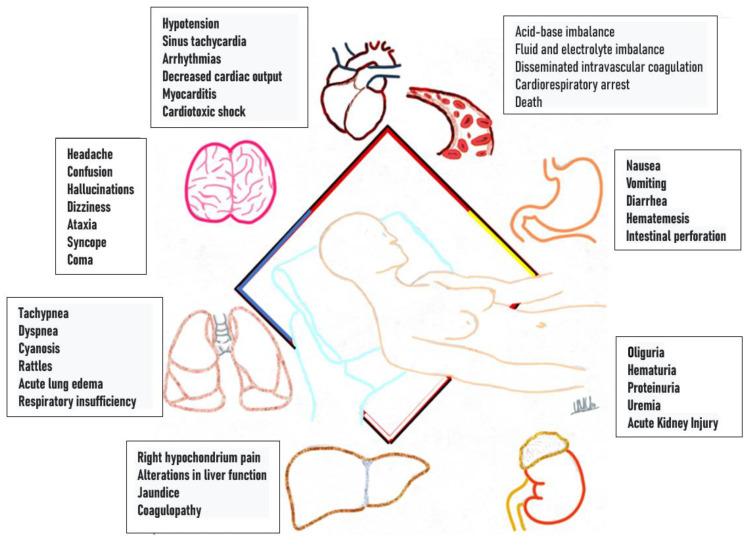
Clinical manifestations by system.

The clinical manifestations are due to multi-organ toxicity [7], secondary to cellular hypoxia to different organs and systems: gastrointestinal, respiratory, cardiac [17], hepatic and others, such as the pancreas and adrenal glands (Figure 8). It leads to disseminated intravascular coagulation, electrolyte and acid–base disorders [14,17]. Postmortem damage to different organs with high oxygen demand can be evidenced: heart, lung, kidney and liver [6], such as pulmonary edema, lesions due to asphyxia in the lung parenchyma, congestion of gastrointestinal mucosa, and cerebral and hepatic petechial hemorrhages [2].

### 3.1. Decontamination Measures and Absorption Reduction

The safety of medical and nursing personnel with adequate personal protective equipment is mandatory given that phosphine gas is released quickly and easily. The appropriate equipment consists of respiratory protection with a full-face mask with positive pressure since the concentration is unknown and mucotegumentary protection with gloves and a gown. Although absorption through the skin and eyes has not been frequently reported [6,7,24], these good practices allow health workers to work under safe conditions. Isolation and a ventilated area [25] are indicated, and thus, the patient must be transferred to a space with these characteristics to avoid secondary contamination. Contaminated clothing should be removed and bagged. In the Toxicological Service of the Hospital Juárez de México, we have an isolated room that has an extraction hood.

To decrease the absorption of the xenobiotic in the exposed patient, gastric lavage and activated charcoal are described as effective measures. These should not be done with water because it would favor the formation of phosphine; therefore, the literature reports performing gastric lavage with coconut oil, potassium permanganate (1:10,000) or liquid paraffin, considering that stomach acid stimulates the conversion to phosphine gas [7,14]. Gastric lavage with coconut oil in combination with sodium bicarbonate is suggested to decrease the conversion of phosphide to phosphine since the acid increases this conversion [19]. It was reported that the administration of coconut oil 6 h after the ingestion of a lethal dose of phosphide shows a reduction in the expected amount absorbed [2,6,26]; therefore, it is proposed as an efficient and effective measure and part of the recommended treatment [6,27]. Coconut oil is high in saturated free fatty acids [19]. The mechanism of action of coconut oil is not clear, but it can form a layer around the gastric mucosa [14,26] that inhibits the release of phosphine gas due to its physicochemical properties and immiscibility with fats [14,19] and it was also described to accelerate gastrointestinal motility and help to remove the toxic compound from the digestive tract [7]. Activated charcoal in a single dose in the first hour after ingestion is recommended due to it having an adsorption action on phosphine [7] and it was shown that its effectiveness decreases over time; the dosage is given according to the age group: neonates and infants from 0 to 12 months old: 10–25 g or 0.5 g–1 g/kg, children from 1 to 12 years old: 25–50 g or 0.5 g–1 g/kg, and adults: 25–100 g or 1 g/kg. The right candidate must be selected to avoid complications and by considering the contraindications of an unprotected airway and altered neurological status since medical conditions can be further compromised [28]. Potassium permanganate at a dose of 200 mL is described as being able to oxidize phosphine gas to phosphate, thereby reducing the amount of toxic gas, but because it is a strong oxidizing agent, it can cause hemolysis and methemoglobinemia [7]; therefore, we do not recommend it.

In the clinical case presented, gastric lavage was performed 7 h after ingestion, with a mixture of 200 mL coconut oil (melted in a water bath) and 50 cc 8.4% sodium bicarbonate [2,17] (per aliquot) in a single dose via an 18 Fr nasogastric tube.

### 3.2. Initial Approach of Poisoned Patient

The treatment options are very limited [18] and there is no specific antidote [7,17,18], and thus, it is very important to make an early diagnosis [2,29] and start treatment as soon as possible [30]. As with all patients admitted to an emergency department, the first thing to assess is the toxicological survival chain [31], which includes the medical approach to the airway, ventilation and circulation (ABC). In the scenario of a voluntarily intoxicated patient, 16% will require intubation due to presenting with neurological alterations and loss of airway protection reflexes; the anticipated risk of bronchial aspiration or as management of intoxication related to crises seizures, severe agitation or delirium; or ventilatory or oxygenation failure [32]. They usually require advanced airway management and invasive ventilation with positive pressure due to acute respiratory distress syndrome, refractory shock, hypoperfusion-induced coma, hemodynamic instability [32] or acute pulmonary edema, which may occur secondary to pulmonary vasodilatation and alveolar fluid extravasation [17,33]. Therefore, we do not rule out early airway protection in the setting of an intoxicated patient with a predictably severe course. It should be taken into account that cyanosis that does not respond to oxygen therapy may be a sign of methemoglobinemia and, if confirmed, requires treatment with methylene blue (1% solution) at a dose of 2 mg/kg over 5 min. Installation of a peripheral venous line or central venous access (if required), fluid resuscitation with crystalloid solutions and vasopressors may be required in appropriate circumstances [7]. The administration of sodium bicarbonate as an adjuvant treatment and its benefit in metabolic acidosis is controversial considering that the main cause of metabolic acidosis secondary to this xenobiotic is a consequence of tissue hypoperfusion, and thus, it should be limited mainly to pH < 7.0 [7,34]. Hemodialysis should be considered in the case of fluid overload and renal failure [14].

### 3.3. Support Measures

The toxic effects of phosphine on myocytes, the adrenal gland and fluid loss can induce circulatory collapse [2], as well as produce refractory shock [6,7,14,23]. Therefore, minimally invasive continuous hemodynamic monitoring [14] and echocardiography can help to guide treatment [35]. Despite the fact that the intravenous administration of fluids is ubiquitously recommended [35], we recommend that it be measured, ensuring adequate perfusion [7], since considering the pathophysiology of the loss of the integrity of the vascular wall and congestion of vital organs, the liquids that are administered diluted with the drugs must also be quantified to avoid fluid overload and complications, such as acute pulmonary edema [7,33]. In 2017, in a systematic review of the literature [35], Cassandra A. et al. reported that in the setting of an intoxicated patient, animal studies generally do not support the use of vasopressors, even suggesting that they are harmful with increased mortality, and there are only case reports in humans that suggest they are often ineffective, although not necessarily harmful. Norepinephrine and epinephrine are the initially recommended options; dopamine and dobutamine are vasoactive agents with greater affinity for beta receptors and are, therefore, associated with a greater arrhythmogenic potential [7]. Cardioversion, the use of a temporary pacemaker or the installation of a balloon pump, and veno-arterial extracorporeal membrane oxygenation (VA ECMO) therapy for mechanical circulatory support should be considered as therapeutic options in patients with cardiotoxic shock this is refractory to vasopressors [7,14,16,36], and in medical units where the resource is available [1,33]. Therefore, the rapid recognition of severity and decision-making before the onset of the deterioration in ventricular function are essential to produce a good prognosis [7]. Steroids in refractory shock are not recommended within the therapeutic links, and stress doses should be started only if adrenocortical insufficiency is documented with decreased serum cortisol levels; however, it is not routinely recommended [7]. Other techniques include continued renal replacement therapy (CRRT), such as hemodiafiltration and hemoperfusion [7], where the latter was used successfully in our hospital unit but is the subject of another review.

### 3.4. Antioxidants

Oxidative stress and the production of free oxygen radicals (ROS) mediate the mechanism of phosphine toxicity; therefore, using antioxidants such as N-acetylcysteine, glutathione, melatonin, vitamin C, vitamin E and carotenes [2] may be beneficial and limit toxicity [6]. Experiments in rats concluded that N-acetylcysteine reduces phosphide-induced myocardial oxidative damage [6], replenishing cellular glutathione [14,25,37], and improves hepatic manifestations and prevents hepatic necrosis [2]. In 2018, a case where a woman was poisoned with zinc and aluminum phosphide was reported; she survived due to treatment based on NAC, digoxin and insulin [6,38]. In 2013, a prospective, randomized and controlled study was carried out with 36 patients, where a reduction in hospitalization time, mortality and the use of mechanical ventilation assistance was observed, confirming that NAC with a loading dose of 150 mg/kg in one hour, followed by 50 mg/kg in 4 h and 100 mg/kg in 16 h, alleviated aluminum phosphide poisoning [6,39]. We used the scheme administered orally, with a loading dose of 140 mg/kg and a maintenance dose of 70 mg/kg for 17 doses [39].

### 3.5. Magnesium

Myocytes are highly susceptible to oxidative stress, which leads to a reduced ejection fraction of the left ventricle and, consequently, to heart failure. Therefore, the correction of electrolyte alterations and the acid–base balance that limits the arrhythmogenic potential must be a priority and immediate [7]. The medical literature is not conclusive regarding the use of magnesium in these patients since it was reported that regarding phosphide poisoning, it does not induce hypomagnesemia [6,7,40], but other investigations showed that serum magnesium levels decreased [14,41] and others showed a reduction in mortality associated with the administration of magnesium in aluminum phosphide poisoning [41]; therefore, this electrolyte must be taken into account. Considering that phosphide intoxication induces oxidative stress, which increases lipid peroxidation and reduces glutathione levels, with the consequent magnesium-dependent drop in magnesium and glutathione levels, it is understandable that after starting magnesium sulfate (MgSO4) therapy, improvement was reported [6,7] due to its membrane stabilizerand antioxidant effects [6,7,14,19,20,21,22,42,43]. There were further reports of mortality reduction above 50% [14,25,41] and survival improvement in a case–control study that compared the use and non-use of this therapeutic link [14,20]. There are different regimens: (1) infusion of 3 g in 3 h, followed by an infusion of 6 g every 24 h for 3 to 5 days; (2) loading dose of 1 g, then 1 g per hour for the next two hours, followed by 1 to 1.5 g every 6 h for 5 to 7 days; (3) loading dose of 4 g, followed by 2 g after one hour and then 1 g every 3 h; and (4) loading dose of 3 g, followed by an infusion of 6 g every 12 h for 5 to 7 days [7,44]. In this case, MgSO4 1 g IV was administered as a bolus and subsequently 1 g per hour for 24 h, and subsequently 1 g every 6 h for 5 days, and our patient did not present clinical or biochemical alterations of hyper- or hypomagnesemia, where the serum level values were in the low normal limit (1.6 and 1.7 mg/dL), and thus, it cannot be ruled out that there was indeed serum Mg depletion.

### 3.6. Hyperinsulinemia–Euglycemia Therapy

Insulin has been known to have positive cardiac inotropic properties since the 1930s, although the mechanism is not fully understood. The use of high doses of insulin together with glycemic support, also known as hyperinsulinemia–euglycemia therapy (HIET), was initially used in toxicology in poisoning beta-blockers and calcium antagonists [17,18,45]. In 2008, it was introduced as a possible treatment for aluminum phosphide poisoning by Hassanian-Moghaddam [18,46].

For a toxin such as phosphine, which induces myocardial depression in up to 60% to 100% of cases [23], the utility of HIET can restore the hemodynamic state to normal, increasing cardiac contractility and improving tissue perfusion [42].

In the absence of stress, the heart primarily catabolizes free fatty acids to obtain its energy needs [7,28,33,45]. In a critical scenario, such as shock and phosphine poisoning, the stressed myocardium shifts its metabolic pathway from b-oxidation to glycolysis [7,18,33], restoring calcium flux [17]. The liver responds to stress, making more glucose available through glycogenolysis, which leads to hyperglycemia.

HIET provides metabolic support to the alterations associated with cardiotoxic shock [42]. It increases both glucose uptake and pyruvate dehydrogenase activity, which improves lactate oxidation, limiting metabolic acidosis [18]. It increases the calcium-dependent ATPase activity in the sarcoplasmic reticulum, which increases the cytoplasmic calcium concentration and improves the calcium entry into mitochondria in animal models [47]. In addition, it decreases the circulation of free fatty acids and promotes the reuptake of intracellular glucose; consequently, it restores the activity of the ionic channels of the membrane and the cell viability of myocytes [7]. The vasodilator property of insulin can occur in systemic, coronary and pulmonary vasculature, and thus, it is not considered a vasopressor. The mechanism is probably due to the activation of the enzyme inositol triphosphate kinase (PI3K), which increases the activity of endothelial nitric oxide synthase. Coronary vasodilation makes it possible to improve tissue perfusion, thus improving cardiac contractility [42]. In a prospective study from 2016 to 2017 by Pannu et al. [17] in a hospital in northern India, they showed a reduction in the need for mechanical ventilation with the use of high-dose insulin infusion, as well as an increase in survival [17]. The inotropic effect of insulin occurs because it metabolically supports the heart during hypodynamic shock, demonstrating the direct correlation between myocardial glycolytic activity and increased cardiac index [42].

When regular insulin is administered intravenously, an immediate peak plasma concentration is obtained. The onset and peak of the cardiovascular effects of insulin occur between 15 to 40 min after administration [33,42,47], and the duration of the effect is 3 to 6 h [17]. Insulin has a volume of distribution of 2 L and protein binding of 5%. More than 50% is metabolized in the liver and is also metabolized in adipose tissue and muscle. The reported adverse effects of high doses of insulin are hypoglycemia, hyperglycemia, hypokalemia, hypomagnesemia and hypophosphatemia [17,42,47]. However, if they occur, it is not an indication to suspend treatment [33] since it restores them to their normal values and adversity is resolved; therefore, they should be closely monitored [33,45,48] by measuring serum phosphate and magnesium every 6 h [47]. Both serum electrolytes and blood glucose in this patient were replaced as required.

Rapid-acting insulin was used at a concentration of 10 international units (IU) for every 1 milliliter of 0.9% saline solution [33], that is, in a 10:1 ratio; the glucose supply was achieved via a 50% glucose solution since selecting a 50% concentration reduces the administration of intravenous fluids [45] because these patients often receive large amounts of fluid [45]. The administration of these solutions requires using a central venous catheter (to avoid peripheral venous thrombophlebitis) and through different lumens [33,47].

The initial dose of insulin is 1 IU/kg in bolus and the glucose supply is calculated at 0.5 g/kg [33,47]; if glucose is less than 200 mg/dL, an initial bolus of glucose solution (1 mL/kg of solution glucose 50%) should be previously administered [33,45]. Insulin infusion should continue from 0.2 IU/kg/h to 1 IU/kg/h [7,42,47] up to a maximum of 10–25 IU/kg/h, with increments of 0.5–1 IU/kg/h every 15 min while considering hemodynamic stabilization [33,47], with an intravenous glucose supply with 50% glucose solution [47] to maintain ranges between 100 and 250 mg/dL [4,14,38], and strict monitoring of electrolytes, allowing for a serum potassium level of 2.8 to 4.5 mmol/L [17,33]. When starting this treatment, it is necessary to monitor serum glucose every 15–30 min; after the glycemia goals are maintained, monitoring will be less frequent, spacing it every hour and then every 4 h [47].

Hemodynamic monitoring should be performed for the cardiac ejection fraction and cardiac contractility [7,47]. The following therapeutic goals are considered to reduce and suspend therapy: hemodynamic stability, mean arterial pressure > 60–65 mmHg, systolic blood pressure > 90 mmHg, sinus rhythm, urinary output of 1–2 mL/kg/h, and eradication of lactate acidosis or lactate < 2 mmol/L [33,45].

In the clinical case presented, therapy was started with a bolus of insulin at a dose of 1 IU/kg and a maintenance dose of 1 IU/kg/h. Capillary glucose was monitored every 20 min until the targets were reached and hourly thereafter. The maximum insulin infusion dose was 7 IU/kg/h at 8 h from the start of treatment. Dose reduction began at hour 72 of treatment and was discontinued at 78 h after initiation. A capillary glucose target between 100 and 250 mg/dL was maintained. The glucose supply began at 0.5 g/kg, with a dose adjustment of 0.7 g/kg, and withdrawal was achieved 22 h after stopping the insulin therapy. Indirect biochemical variables of tissue perfusion were measured, such as pH, lactate and bicarbonate, as well as a close clinical evaluation, observing improvement all around (Figure 2, Figure 3 and Figure 4).

#### Reduction in Hyperinsulinemia–Euglycemia Therapy

According to the literature, the duration of therapy with high doses of insulin lasts from 9 to 49 h, with an average of 27 h [48]. The withdrawal of intravenous glucose supply is recommended after the suspension of hyperinsulinemia therapy. It usually lasts from 24 [48] to 48 h [29,49] or between 9 and 72 h, with a mean of 47 h [48], since the half-life of insulin is substantially prolonged after an infusion of 10 IU/kg/hour [29]. In a case report in 2020, Corcoran et al. verified [50] that serum insulin levels remain at supraphysiological levels after the use of therapy of a high dose of insulin at 10 IU/kg/h with a total duration of 37 h (observing a reduction in the dose of norepinephrine during therapy), considering that a total interruption of insulin could be allowed in place of a gradual decrease at the end of therapy.

**Figure 9:**
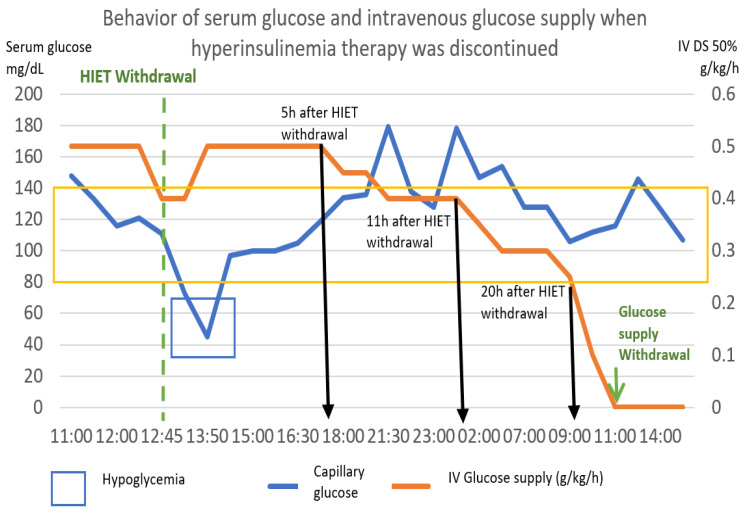
She response of capillary glucose and intravenous glucose supply was observed, after withdrawal of hyperinsulinemia–euglycemia therapy. It was observed that the decrease in the contribution of glucose to the withdrawal of insulin produced hypoglycemia, though we managed to remove this contribution up to 22 h later. The numerical scale and units of measurement of Serum glucose, illustrated with the blue line, are plotted on the left-hand “*y*”-axis. The numerical scale and units of measurement of intravenous glucose supply, illustrated with a orange line, are plotted on the right-hand “*y*”-axis.

The following graph (Figure 9) shows the behavior of capillary glucose and the glucose supply after the withdrawal of hyperinsulinemia therapy.

Based on the pattern shown by this patient, the following scheme was proposed as a point of good practice, with capillary glucose monitoring every 2 h. In our case, 5 h after the withdrawal of hyperinsulinemia therapy, the intravenous glucose intake was reduced by 0.1 g/kg. Six hours later, it was reduced again by 0.1 g/kg, and 9 h later, it was reduced again by 0.1 g/kg. After 20 h post discontinuation of high-dose insulin, it was reduced by 0.1 g/kg/hour per hour, achieving cessation at 22 h after the initiation of withdrawal.

### 3.7. Intravenous Lipid Emulsion

Intravenous lipid emulsion (ILE) was introduced in 1960 as an energy substrate because it contains essential fatty acids, and it was administered parenterally as a nutritional supplement in seriously ill patients [51,52]. Its effectiveness was also demonstrated in 2006 in local anesthetic toxicity, such as bupivacaine, and later by fat-soluble xenobiotics with different toxicokinetic and mechanisms of action [51,52]. In 2010, Zhou et al. [53] proposed ILE in combination with hemoperfusion with carbon filters in patients poisoned by organophosphates.

ILE’s mechanism of action in phosphine intoxication is not defined, and neither is its mechanism of action with some water-soluble xenobiotics [52]. However, several mechanisms are described: (1) The original theory of a “lipid sink”, which is based on the high lipid solubility of the toxicant [52], where it sequesters lipophilic compounds in an expanded intravascular lipid phase, allowing the lowest serum concentration to generate a concentration gradient, removing the tissue agent and reducing its concentration, effect and toxicity at the site of action and in critical organs, such as heart and brain [7,26,33,51,52,53,54]. (2) A metabolic effect by increasing the energetic substrate to the myocyte [52,53] and a direct cardiotonic effect on cardiotoxicity, improving cardiac function. (3) The cardioprotective action of long-chain fatty acids in ILE was found to involve Ca^2+^ homeostasis and rescue signaling pathways that regulate the opening of the mitochondrial permeability transition pore (mPTP), which restores mitochondrial Ca^2+^ levels [51].

A series of cases of aluminum phosphide poisoning were published in countries such as India [2,6,17,30,55,56,57,58,59,60,61,62], where it is a common cause of occupational accidents in the agricultural community and voluntary oral exposure with suicidal intentions [2,17]; these cases involved the administration of ILE 20%, recommended dose of 10 mL/hour with monitoring of triglycerides [26]. There are different administration schemes for ILE 20%: (1) a loading dose of 1–1.5 mL/kg, followed by infusion at 10 mL/hour [26]; (2) a loading dose of 1.5 mL/kg, followed by infusion of 0.25–0.5 mL/kg/min [33,63]; and (3) a loading dose of 1.5 mL/kg, followed by 0.25 mL/kg/min for 3 min and then an infusion of 0.025 mL/kg/min for 6.5 h. The infusion should be stopped at the completion of the maximum dose (10–12 mL/kg/d or a maximum of 10% of the total blood volume, especially in obese, neonate and young patients), the maximum duration or the resolution of toxicity. However, there is no routinely recommended dose or sufficient evidence to indicate when to stop the infusion [63].

The clinical and biochemical response showed improvement with ILE 20% (Figure 5, Figure 6 and Figure 7); at the same time, vigilance was maintained for possible adverse effects, such as hematologic effects (intravascular hemolysis, disseminated intravascular coagulation), renal effects (acute kidney injury), pulmonary effects (hypoxia, acute lung injury, respiratory distress syndrome), acid–base imbalance effects (metabolic acidosis), vascular effects (deep vein thrombosis, phlebitis), hypersensitivity and allergic adverse effects, fat overload syndrome (hypertriglyceridemia, lipemia, hyperamylasemia, pancreatitis, cholestasis) and immunological effects (susceptibility to infections) [64]. In our patient, there were no short-term complications.

### 3.8. Other Therapeutic Strategies

A range of options were described for when cardiotoxic shock occurs, such as:

Digoxin, which would increase cardiac contractility and blood pressure, at a dose of 0.5 mg every 6 h for one day and subsequently 0.25 mg every 24 h [2,48,65].

Trimetazidine, which inhibits 3-ketoacyl CoA thiolase in the mitochondrial matrix and catalyzes fatty acids into acetyl CoA for its entry into the Krebs cycle and consequent production of ATP, thus preventing the decrease in intracellular ATP. It inhibits the oxidation of fatty acids and favors the oxidation of glucose, ensuring the functioning of ionic pumps and the transmembrane flow of sodium and potassium, and maintaining cell homeostasis. The suggested dose is 20 mg to 35 mg every 12 h [7,14].

#### Other Drugs Described in the Literature

Melatonin has an amphiphilic structure, which penetrates the mitochondria. Various benefits are described for it: it prevents apoptosis by restricting mitochondrial pore permeability and disturbing caspase activation, intensifies the production of ATP, weakens the suppression of the respiratory chain and it has antioxidant effects.

Levothyroxine decreases the activity of caspases 3 and 9, increasing cell viability, at a recommended dose of 3 mcg/kg.

Taurine, which enhances the detrimental effects of phosphine on the electron transport chain, enhances mitochondrial membrane potential and reduces oxidative stress.

Boric acid, which is recognized as a Lewis acid, and thus, it has an empty p orbital, which can accept electrons, and phosphine offers electrons to cytochrome C oxidase, interfering with the mitochondrial respiratory chain; therefore, phosphine reacts with boric acid and produces a gaseous adduct, where the frequency of gas evolution decreases but not the volume of release.

Other drugs with an antioxidant function are as follows:

Vitamin E at a dose of 100 U every 12 h for 72 h.

Vitamin C at a dose of 1 g every 8 h in the first 24 h.

However, neither of these have a routine recommendation [7].

## 4. Analysis of Therapeutic in a Real Clinical Scenario

In this case, the therapeutic approach was decided in a stepwise manner due to the lack of clinical improvement and the worsening of tissue hypoperfusion biomarkers during the first hours of management.

From the beginning, management with antioxidant therapies and electrolyte support with magnesium was indicated. N-acetylcysteine was administered to protect against fostin-induced myocardial oxidative damage and to restore intracellular glutathione levels. Likewise, with the aim of preventing hepatic necrosis, electrolyte support with magnesium sulfate was initiated from the beginning, considering that phosphine induces oxidative stress, which increases lipid peroxidation and reduces glutathione levels, with a consequent decrease in magnesium and magnesium-dependent glutathione. The literature reports a mortality reduction effect of over 50% using this strategy, which indicates its importance. Its effects are synergistic with N-acetylcysteine in restoring glutathione and magnesium-dependent glutathione levels. At the same time, management with HIET was started, providing metabolic and cardiovascular support. Considering its vasodilator effect at the coronary level, it prevents myocardial hypoperfusion and progression to cardiotoxic shock. After the first 3 h of HIET, an increase in lactate from an initial value of 7.7 to 8.4 mmol/L was observed, accompanied by a decrease in pH from 7.29 to 7.22, as well as a decrease in serum bicarbonate from 10.5 to 5.1 mmol/L. Furthermore, at the clinical level, a drop in mean blood pressure from 93 to 76 mmHg was observed. Therefore, ILE was indicated as a second antidotal line to provide a means of redistribution of the xenobiotic and reducing its toxic effects, and thus, provide stability to the mitochondrial membrane and maintain ATP production in the myocardium.

Two and a half hours after initiation, the pH increased from 7.22 to 7.26, bicarbonate increased from 5.1 to 9.8 mmol/L and serum lactate dropped from 8.4 to 5.6. Ten hours after the start of ILE, a pH of 7.42, bicarbonate of 17.2 and lactate of 2.4 were observed. During the evolution, no vasopressor support was required and no evidence of cardiac failure was observed. Moreover, bicarbonate replacement was not required.

This behavior supports the theory that the vasoregulatory and inotropic effects of insulin in conjunction with the hyperglycemic substrate of HIET provide metabolic support to the myocardium, preventing it from developing cardiotoxic shock, even in this case in which the ingested dose exceeded the proposed lethal dose 50 for humans. Regarding the possible positive effects of the use of ILE in this case, it was contemplated that the cardioprotective action of long-chain fatty acids in ILE involves Ca^2+^ homeostasis and rescue signaling pathways that regulate the opening of the mitochondrial permeability transition pore (mPTP), which restores mitochondrial Ca^2+^ levels, i.e., a synergistic effect between the inotropic effects of HIET and ILE was contemplated. Although the broader recommendations in the medical literature support the use of regular insulin to institute HIET therapy, it was noted in this case that the use of rapid-acting insulin is equally safe and effective.

**Figure 10:**
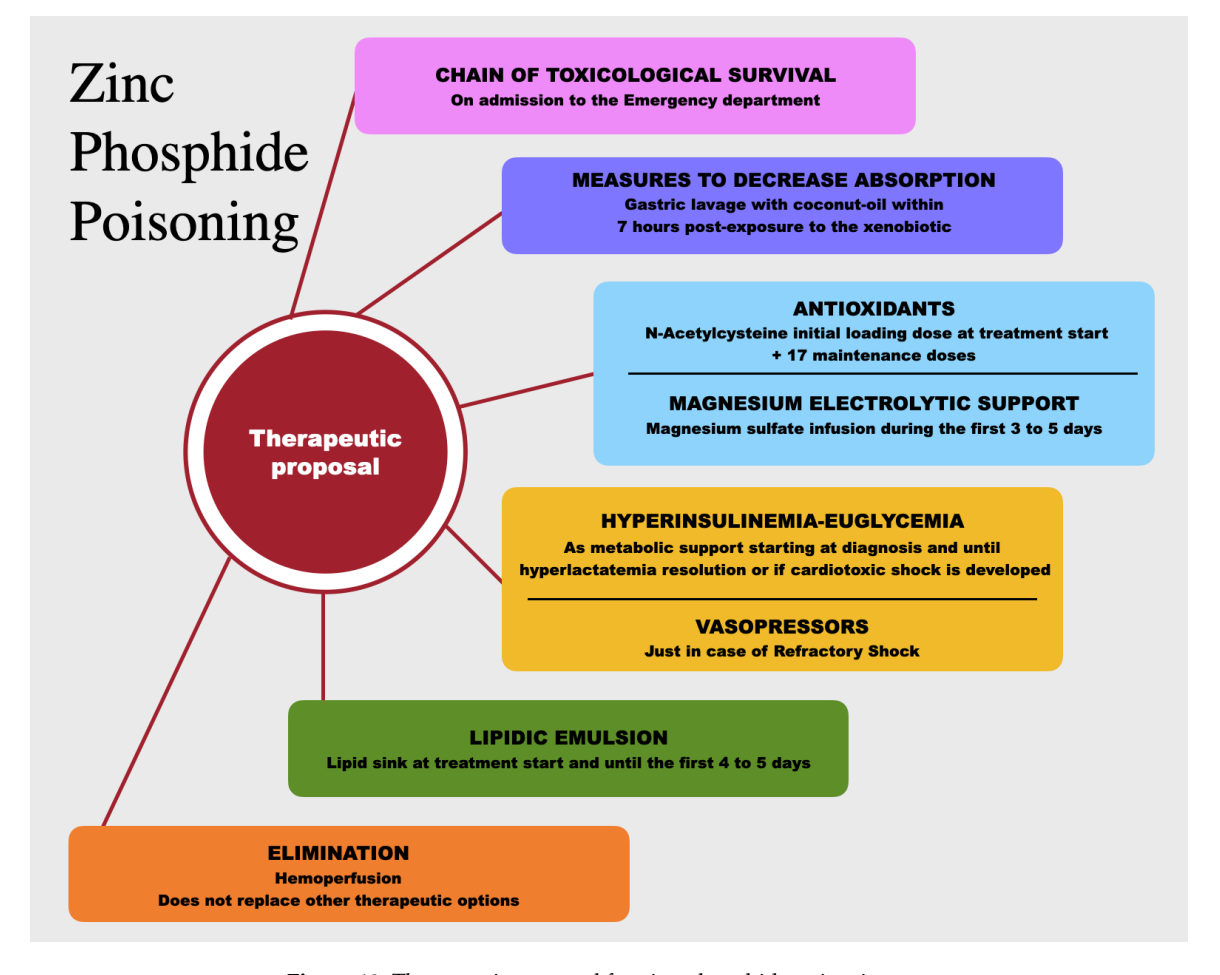
Therapeutic proposal for zinc phosphide poisoning.

Taking this into consideration, the following therapeutic proposal was made. We suggest early initiation of support with magnesium sulfate and N-acetylcysteine, together with EILH and ILE in a staggered manner. The use of vasopressors and inotropic support should be reserved only for the case of evolution to cardiotoxic shock (Figure 10).

## 5. Conclusions

Zinc phosphide poisoning is highly lethal and there is no specific antidote; even the literature is controversial. It can occur accidentally or voluntarily due to suicidal intentions. Despite the lack of international guidelines for its care, the understanding of its pathophysiological bases allows for an adequate approach that, through current therapeutic strategies, not only allows for increasing survival but also opens the door to further research in terms of both the mechanisms of action of each therapeutic resource and the development of better therapeutic options. Herein, we present a case of ingestion of zinc phosphide with suicidal intent at a dose above the LD50, which responded adequately to our stepwise therapy proposal in which early initiation of therapy surely increased survival.

The first step for the first-contact physician is not to fear exposure since there is adequate personal protective equipment to provide immediate and high-quality care; therefore, emergency medical services must have the necessary material and human resources.

## Data Availability

The reported results are supported in the clinical record, stored in the Clinical Archive of the Hospital Juárez de México. There is no electronic availability.

## References

[1] Çakın Ö., Tazegul G., Gümüş A., Cengiz M., Ramazanoğlu A. (2018). Incidental Aluminum Phosphide Poisoning: Case Report and Current Management. Folia Med..

[2] Moghadamnia A. (2012). An update on toxicology of aluminum phosphide. DARU J. Pharm. Sci..

[3] (1991). Catálogo Oficial de Plaguicidas 1991.

[4] Fernando Bejarano González Los Plaguicidas Altamente Peligrosos en México, Primera edición, julio 2017. Red de Acción Sobre Plaguicidas y Alternativas en México, A.C. (RAPAM). https://www.rapam.org/wp-content/uploads/2017/09/Libro-Plaguicidas-Final-14-agst-2017sin-portada.pdf.

[5] Comisión Federal Para la Protección Contra Riesgos Sanitarios (*COFEPRIS*) Plaguicidas 2015. http://www.cofepris.gob.mx/AZ/Paginas/PlaguicidasyFertilizantes/PlaguicidasYFertilizantes.aspx.

[6] Karimania A., Hooshang A., Reza M., Rezaee R., Megabane B., Tsatsakis A., Karimi G. (2018). Antidotes for aluminum phosphide poisoning An update. Toxicol. Rep..

[7] Anbalagan L.C., Arora N., Pannu A.K. (2021). Management of Acute Aluminum Phosphide Poisoning: Has Anything Changed?. Drug Metab. Lett..

[8] Enviromental Health Criteria 73 (1988). Phosphine and Selected Metal Phosphides, IPCS International Programme on Chemical Safety.

[9] Vij K. (2008). Textbook of Forensic Medicine and Toxicology-Principles and Practice.

[10] Gurvinder S.B. (2012). Phosphide poisoning: A review of literature. Forensic Sci. Int..

[11] Sánchez M., Bárcena A. (2017). Intoxicación con fosfuro de zinc en el paciente pediátrico en un centro toxicológico de la Ciudad de México. Rev. Médica Inst. Mex. Seguro Soc..

[12] López Islas I., Juan J., Nuevo L. (2008). Edema Agudo Pulmonar No Cardiogénico en Pacientes con Intoxicación por Fosfuro de Cinc. Reporte de dos Casos y Revisión Bibliográfica. Med. Intern. Mex..

[13] Watson W.A., Litovitz T.L., Klein-Schwartz W., Rodgers G.C., Youniss J., Reid N., Youniss J., Flanagan A., Wruk K.M. (2004). Annual report of the American Association of Poison Control Centers Toxic Exposure Surveillance System. Am. J. Emerg. Med..

[14] Mehrpour O., Jafarzadeh M., Abdollahi M. (2012). A Systematic Review of Aluminium Phosphide Poisoning. Arch. Ind. Hyg. Toxicol..

[15] Pérez Navero J.L., Ibarra de la Rosa I., Frías Pérez M.A., Arroyo Marín M.J., Pérez Jorge P. (2009). Intoxicación letal por inhalación accidental de fosfuro alumínico. An. Pediatría.

[16] Mehrpour O., Asadi S., Yaghoubi M.A., Azdaki N., Mahmoodabadi N., Javadmoosavi S. (2019). Cardiogenic Shock Due to Aluminum Phosphide Poisoning Treated with Intra-aortic Balloon Pump: A Report of Two Cases. Cardiovasc. Toxicol..

[17] Pannu A., Bhalla A., Gantala N., Shafrma S., Kumar S., Dhibar D. (2020). Glucose-insulin-potassium infusion for the treatment of acute aluminum phosphide poisoning: An open-label pilot study. Clin. Toxicol..

[18] Hassanian-Moghaddam H., Zamani N. (2016). Therapeutic role of hyperinsulinemia/euglycemia in aluminum phosphide poisoning. Clin. Trial/Exp. Study Med..

[19] Shadnia S., Rahimi M., Pajoumand A., Rasouli M.-H., Abdollahi M. (2005). Successful treatment of acute aluminium phosphide poisoning:Possible benefit of coconut oil. Hum. Exp. Toxicol..

[20] Sasser S.M., Viccellio P., Bania T., Brent J., Hoffman R.S., Kulig K.W., Mofenson H.C., Osborn H.H., Wang R.Y., Wax P.M. (2019). Rodenticides. Emergency Toxicology.

[21] Chugh S.N., Kolley T., Kakkar R., Chugh K., Sharma A. (1997). A critical evaluation of anti-peroxidant effect of intravenous magnesium in acute aluminium phosphide poisoning. Magnes. Res..

[22] Chugh S.N., Arora V., Sharma A., Chugh K. (1996). Free Radical Scavengers & Lipid Peroxidation in Acute Aluminium Phosphide Poisoning. Indian J. Med. Res..

[23] Petrovic M., Otero D., Leigh A., Singh V. (2021). Acute Hearth Failure Due to Aluminium Phosphide Poisoning. Methidist DeBakey Cardiovasc. J..

[24] Sudakin D.L. (2005). Occupational exposure to aluminium phosphide and phosphine gas? A suspected case report and review of the literature. Hum. Exp. Toxicol..

[25] Bogle R.G. (2006). Aluminium phosphide poisoning. Emerg. Med. J..

[26] Udismita B., Ameeta S., Harish S. (2015). Successful management of aluminium phosphide poisoning using intravenous lipid emulsion: Report of two cases. Indian J. Crit. Care Med..

[27] Bajwa S.S., Bajwa Kaur S., Kaur J., Singh K., Panda A. (2010). Management of celphos poisoning with a novel intervention: A ray of hope in the darkest of clouds. Anesth. Essays Res..

[28] Chyka P.A., Seger D., Krenzelok E.P., Vale J.A. (2005). Position Paper: Single-Dose Activated Charcoal. American Academy of Clinical Toxicology & European Association of Poisons Centers and Clinical Toxicologists. Clin. Toxicol..

[29] Corcoran J.N., Jacoby K.J., Olives T.D., Bangh S.A., Cole J.B. (2021). In Reply: “On Insulin Kinetics Following High-Dose Insulin Therapy, and When to Stop Therapy”. J. Med. Toxicol..

[30] Saraswat P.K., Gupta B.P., Malhotra V.K., Goyal V.K. (1985). Prevalence of fatalities due to aluminium phosphide poisoning in Southern Rajasthan (an epidemiological study). J. Forensic Med. Toxicol.

[31] Dorado García R., Soto Estrada M., Ontiveros Holguín A. (2022). 10 Errores Graves en el Manejo del Paciente Intoxicado. Crit. Care Emerg. Med..

[32] Burket G., Zane B., Hendrickson R., Beauchamp G. (2020). Endotraqueal Intubation in the Pharmaceutical-Poisoned Patient: A Narrative Review of the Literatura. J. Med. Toxicol..

[33] Stephen V., Pluymers N., Gauton S. (2019). Emergency management of calcium channel blocker overdose. Afr. Med..

[34] Marashi S., Nasri Z. (2015). Can sodium bicarbonate really help in treating metabolic acidosis caused by aluminium phosphide poisoning?. Arh. Hig. Rada Toksikol..

[35] Skoog C., Engebretsen K. (2017). Are vasopressors useful in toxin-induced cardiogenic shock?. Clin. Toxicol..

[36] Abdollahi M., Karami-Mohajeri S. (2012). A comprehensive review on experimental and clinical findings in intermediate syndrome caused by organophosphate poisoning. Toxicol. Appl. Pharmacol..

[37] Azad A., Lall S.B., Mittra S. (2001). Effect of N-acetylcysteine and L-NAME on aluminium phosphide induced cardiovascular toxicity in rats. [Internet]. Acta Pharmacol. Sin..

[38] Aliasghar M., Ahangar R.M., Pedram B., Samaneh N., Omid M. (2018). A case of successful treatment of heart failure due to simultaneous poisoning with aluminum phosphide and zinc phosphide, Iran. Red Crescent Med. J..

[39] Tehrani H., Halvaie Z., Shadnia S., Soltaninejad K., Abdollahi M. (2012). Protective effects of N-acetylcysteine on aluminum phosphide-induced oxidative stress in acute human poisoning. Clin. Toxicol..

[40] Siwach S.B., Singh P., Ahlawat S., Dua A., Sharma D. (1994). Serum & tissue magnesium content in patients of aluminium phosphide poisoning and critical evaluation of high dose magnesium sulphate therapy in reducing mortality. [Internet]. J. Assoc. Physicians India.

[41] Chugh S.N., Kamar P., Sharma A., Chugh K., Mittal A., Arora B. (1994). Magnesium status and parenteral magnesium sulphate therapy in acute aluminum phosphide intoxication. [Internet]. Magnes. Res..

[42] Stellpflug S., Kerns W., Flomenbaum N.E., Goldfrank L.R. (2006). High-Dose Insulin. Goldfrank’s Toxicologic Emergencies.

[43] Dua R., Gill K.D. (2001). Aluminium Phosphide Exposure: Implications on Rat Brain Lipid Peroxidation and Antioxidant Defense System. Pharmacol. Toxicol..

[44] Chugh S.N., Kumar P., Aggarwal H.K., Sharma A., Mahajan S.K., Malhotra K.C. (1994). Efficacy of magnesium sulphate in aluminium phosphide poisoning--comparison of two different dose schedules. J. Assoc. Physicians India.

[45] Krenz J., Kaakeh Y. (2018). An Overview of Hyperinsulinemic-Euglycemic Therapy in Calcium Channel Blocker and β-blocker Overdose. Pharmacotherapy.

[46] Hassanian-Moghaddam H. (2008). NACCT Abstracts of the 2008 North American Congress of Clinical Toxicology Annual Meeting, Toronto, ON, Canada, 11–16 September 2008.

[47] Hamzic J., Raos D., Radulovic B. (2022). High-Dose Insulin euglucemic therapy. Acta Clínica Croac..

[48] Engebretsen K.M., Kaczmarek K.M., Morgan J., Holger J.S. (2011). High-dose insulin therapy in beta-blocker and calcium channel-blocker poisoning. [Internet]. Clin. Toxicol..

[49] Gawedzki P., Paloucek F.P. (2021). Additional Considerations for Persistent Hyperinsulinemia. J. Med. Toxicol..

[50] Corcoran J.N., Jacoby K.J., Olives T.D., Bangh S.A., Cole J.B. (2020). Persistent Hyperinsulinemia Following High-Dose Insulin Therapy: A Case Report. J. Med. Toxicol..

[51] Eisenkraft A., Falk A. (2016). The possible role of intravenous lipid emulsion in the treatment of chemical warfare agent poisoning. Toxicol. Rep..

[52] Cao D., Heard K., Foran M., Koyfan A. (2015). Intravenous Lipid Emulsion in the Emergency Department: A systemic review of recent literature. J. Emerg. Med..

[53] Zhou Y., Zhan C., Li Y., Zhong Q., Pan H., Yang G. (2010). Intravenous lipid emulsions combine extracorporeal blood purification: A novel therapeutic strategy for severe organophosphate poisoning. Med. Hypotheses.

[54] French D., Smollin C., Ruan W., Wong A., Drasner K., Wu A.H. (2011). Partition constant and volume of distribution as predictors of clinical efficacy of lipid rescue for toxicological emergencies. Clin. Toxicol..

[55] Chaudhary S. (2013). An Epidemiological Study of Fatal Aluminium Phosphide Poisoning at Rajkot. IOSR J. Pharm..

[56] Kordrostami R., Akhgari M., Ameri M., Ghadipasha M., Aghakhani K. (2017). Forensic toxicology analysis of self-poisoning suicidal deaths in Tehran, Iran; trends between 2011–2015. DARU J. Pharm. Sci..

[57] Shadnia S., Sasanian G., Allami P., Hosseini A., Ranjbar A., Amini-Shirazi N., Abdollahi M. (2009). A retrospective 7-years study of aluminum phosphide poisoning in Tehran: Opportunities for prevention. Hum. Exp. Toxicol..

[58] Moghadamnia A.A., Abdollahi M. (2002). An epidemiological study of poisoning in northern Islamic Republic of Iran. East. Mediterr. Health J..

[59] Mehrpour O., Singh S. (2010). Rice tablet poisoning: A major concern in Iranian population. Hum. Exp. Toxicol..

[60] Hosseinian A., Pakravan N., Rafiei A., Feyzbakhsh S. (2011). Aluminum phosphide poisoning known as rice tablet: A common toxicity in North Iran. Indian J. Med. Sci..

[61] Hassanian H.M., Pajoumand A. (2007). Two years epidemiological survey of aluminum phosphide poisoning in Tehran. Iran J. Toxicol..

[62] Nosrati A., Karami M., Esmaeilnia M. (2013). Aluminum phosphide poisoning: A case series in north Iran. Asia Pac. J. Med. Toxicol..

[63] Gosselin S., Hoegberg L.C.G., Hoffman R.S., Graudins A., Stork C.M., Thomas S.H.L., Stellpflug S.J., Hayes B.D., Levine M., Morris M. (2016). Evidence-based recommendations on the use of intravenous lipid emulsion therapy in poisoning. Clin. Toxicol..

[64] Hayes B., Gosselin S., Calello D., Nacca N., Rollins C., Abourbih D., Morris M., Nesbitt A., Morais J., Lavergne V. (2016). Lipid Emulsion Workgroup. Systematic review of clinical adverse events reported after acute intravenous lipid emulsion administration. Clin. Toxicol..

[65] Changal K.H., Latief M., Parry M., Abbas F. (2017). Aluminium phosphide poisoning with severe cardiac dysfunction and the role of digoxin. BMJ Case Rep..

